# Knowledge and awareness of the health effects of electronic cigarette among college students

**DOI:** 10.25122/jml-2025-0016

**Published:** 2025-03

**Authors:** Saleh Khateeb, Dana Alsuqaie, Sama Sobahi, Danah Muminah, Laila Alyafi, Taif Alotbi

**Affiliations:** 1 Fakeeh College for Medical Science, Jeddah, Saudi Arabia

**Keywords:** vape, electronic cigarettes, college students, smoking, Jeddah, knowledge, attitude

## Abstract

Electronic cigarettes (e-cigarettes) are nicotine delivery devices promoted as a safer alternative to traditional smoking; however, their long-term health effects remain uncertain. Their use is rapidly increasing worldwide, particularly among young adults. In Jeddah, Saudi Arabia, there is limited information on e-cigarette usage among college students. This study aimed to assess the level of knowledge and awareness regarding the health effects of e-cigarettes among college students in Jeddah through a cross-sectional, survey-based study conducted in 2024 with 438 participants. Data were collected using a pre-validated self-administered questionnaire distributed via various social media platforms. Categorical variables were compared using the chi-square test. Results indicated that 54.8% of respondents reported previous smoking experience. Among the participants, 29.7% believed that e-cigarettes are an effective smoking cessation method, 50.5% viewed them as a potential replacement for traditional cigarettes, and 43.2% considered e-cigarettes a gateway to conventional smoking. Overall, 39.3% of respondents demonstrated good knowledge about e-cigarettes, with significant differences observed between age groups, genders, smoking experience (*P* < 0.001), and educational levels (*P* < 0.002). Additionally, 54.3% of respondents expressed a negative attitude toward e-cigarettes, with statistically significant differences across groups (*P* < 0.001). Given the relatively high prevalence of smoking among college students in Jeddah and their overall insufficient knowledge about e-cigarettes, these findings underscore the urgent need to raise awareness about the risks associated with e-cigarette use and to implement appropriate regulatory measures.

## INTRODUCTION

Smoking is a global health epidemic, with approximately 1.18 billion people using tobacco products and around 7 million deaths attributed to tobacco use in 2020 [1]. In recent years, electronic cigarettes (e-cigarettes) have emerged as a popular alternative to conventional cigarettes, particularly among young adults [2,3]. Globally, the prevalence of vaping among adolescents is estimated to be around 11% [4]. In the Kingdom of Saudi Arabia (KSA), the overall smoking rate was reported at 21.4% in 2013, with 32.5% among men and 3.9% among women, and traditional tobacco cigarettes being the most commonly used form [5]. Despite the official prohibition on the sale of vaping products in Saudi Arabia since September 2015 [6], e-cigarette usage has surged to 26.3% [7]. Furthermore, the composition and effects of e-cigarettes on the population in Saudi Arabia is not currently subjected to any regulations [8].

An e-cigarette is a small electronic device, typically comprising a rechargeable battery, a heating element, and a cartridge filled with e-liquid. The heating element warms the e-liquid—composed of nicotine, flavorings, and other chemicals—producing a vapor that is inhaled through a mouthpiece [9]. The use of e-cigarette has markedly increased probably due to the decline in ordinary cigarette smoking over the past years. Moreover, e-cigarette use among adolescents rose by 10% from 2017 to 2018 and has continued to grow in the following years [10,11].

While early studies suggested that e-cigarettes might aid in smoking cessation [12], more recent evidence indicates that daily e-cigarette users do not experience significant benefits in quitting compared to non-users [13]. Although e-cigarettes generally contain fewer toxicants than conventional cigarettes, the toxin levels can vary significantly among brands [14]. The potential adverse effects of e-cigarettes, due to both nicotine and non-nicotine components, are still under investigation [15]. There is evidence that e-cigarettes can have negative mental health outcomes, including poor academic performance, disrupted sleep, aggressive behavior, attention deficits, depressive symptoms, and suicidal ideation [16].

Although, e-cigarettes may not have immediate cardiac or pulmonary effects [17], they share several hazards with traditional smoking, such as increased respiratory resistance [18]. Additionally, e-cigarette use has been associated with decreased fractional exhaled nitric oxide and alterations in airway immune profiles [19]. The term 'E-cigarette or Vaping Product-Associated Lung Injury' (EVALI) emerged following an epidemic of acute lung injury in over 2,600 cases among users, attributed to significant pulmonary inflammation [20,21]. Furthermore, chronic heart diseases, including increased arterial stiffness, elevated blood pressure, myocardial fibrosis, and coronary vascular disease, have been observed in e-cigarette users [22].

In Jeddah, a 2018 study reported that 27.7% of health science students used e-cigarettes [23]. More recent findings from Saudi Arabia reveal a substantial knowledge gap among adolescent users, many of whom maintain a positive attitude toward these devices. Given the rapid expansion of e-cigarette use over the past five years, there is an urgent need for updated research to better understand current usage patterns and perceptions of e-cigarette safety among university students.

## MATERIAL AND METHODS

### Study design, setting, and population

This cross-sectional study was conducted between January and June 2024 among both undergraduate and postgraduate college students in Jeddah, Saudi Arabia. Participants were recruited using a convenience sampling method, with the survey distributed online via various social media platforms. Announcements outlining the study objectives and including a link to the Google Form questionnaire were posted on Facebook, WhatsApp, X, and Telegram. Interested students received the survey link and were encouraged to share it with their peers.

### Study sample

Based on previous research in Saudi Arabia that reported a 51.1% awareness level and an 80.46% negative attitude toward e-cigarettes [24], and using a 95% confidence interval with a 5% margin of error, we determined that a sample size of 438 college students was required. This calculation was performed using Epi-Info 7 software.

### Study instrument

Participants were provided with a clear explanation of the study’s objectives before being asked to complete an online, self-administered questionnaire available in English and Arabic [25]. The survey aimed to estimate the prevalence of smoking among college students, examine the relationship between knowledge and attitudes toward e-cigarette use and demographic variables (such as age, gender, and educational level), identify factors influencing e-cigarette use among college students in Jeddah (including awareness levels, age preferences, and overall appeal), and evaluate perceptions regarding the addictiveness, safety compared to traditional cigarettes, and the role of e-cigarettes in smoking cessation or initiation.

The questionnaire was structured as follows: Questions 1–3 collected basic demographic data such as sex, age, and academic level. Questions 4–5 assessed previous smoking experience and frequency of smoking. Questions 6–7 gathered information about previous knowledge and sources of information about e-cigarettes. Questions 8 and 9 investigated perceptions and attitudes regarding e-cigarettes and role in smoking cessation. Questions 10–13 evaluated beliefs about the safety and health risks of e-cigarettes compared to conventional cigarettes, including issues related to dependence, secondhand exposure, and cancer risk. Questions 14–15 examined opinions on whether e-cigarettes could replace traditional cigarettes or serve as a gateway to conventional smoking; and Questions 16–18 collected views on the public health implications of e-cigarettes and the importance of regulations controlling their use in public and workplace settings. The questionnaire was revised by experts at the Medical College of Fakeeh College for Medical Science to assess the face validity of the questionnaire, using structural and content validity and clear language.

### Statistical analysis

Data were entered and analyzed using IBM SPSS software version 24.0. Qualitative data were described using numbers and percentages, and the Chi-square test was used to compare categorical variables between different groups. Results were reported as two-tailed *P* values, and a *P* value of <0.05 was considered statistically significant.

### Knowledge and attitude score calculation

Knowledge and attitude scores were calculated as follows. For the knowledge component, each correct answer was assigned 1 point and each incorrect answer 0 points. The total knowledge score was determined by summing these points and then expressing the result as a percentage of the maximum possible score. Knowledge levels were classified as 'good' if the percentage was greater than 65%, 'fair' if it ranged between 50% and 65%, and 'poor' if it was below 50%. For the attitude component, each positive response was awarded 2 points and each negative response 1 point. The total attitude score was similarly calculated by summing the points and normalizing the total as a percentage of the maximum achievable score, with scores greater than 65% considered indicative of a positive attitude, scores between 50% and 65% categorized as neutral, and scores below 50% categorized as negative [3].

## RESULTS

### Participants’ personal and sociodemographic characteristics

A total of 438 completed questionnaires were collected from college students in Jeddah, Saudi Arabia. The participants had a mean age of 21.96 years (range: 18–30 years), with 40.6% men (*n* = 178) and 59.4% women (*n* = 260). In terms of educational attainment, the majority had completed a bachelor’s degree (72.6%), followed by high school graduates (14.4%), master’s degree (10.7%), and PhD (2.3%). Additionally, 54.8% of the respondents reported a smoking history, with 43.8% of smokers indicating an experience of 1–3 years. Among those with a smoking history, 52.1% (*n* = 240) reported smoking fewer than 10 cigarettes per day (Table 1).

**Table 1:** Characteristics of university students at Jeddah, KSA, 2024 (*n* = 438)

	Number	Percent
**Age**		
<20	58	13.2
20-25	165	37.7
>30	215	49.1
Range	18-30	
Mean	21.96	
SD	13.05	
**Sex**		
Men	178	40.6
Women	260	59.4
**Level of education**		
High school	63	14.4
Bachelor	318	72.6
Master	47	10.7
PhD	10	2.3
**Smoking experience**		
Non smoker	198	45.2
Smoker	240	54.8
*For smoker only “n = 240”*		
**Number of years**		
1-3 years	105	43.8
3-6 years	62	25.8
7-10 years	40	16.7
>10 years	33	13.8
**Frequency of smoking (cig/day)**		
<10 cig/day	125	52.1
10-15 cig/day	65	27.1
15-20 cig/day	50	20.8
**Total**	**438**	**100.0**

### Participants’ awareness and knowledge about e-cigarettes

All participants had heard of e-cigarettes, with the most common sources of information being friends (69.9%) and parents (23.3%), followed by newspapers, magazines, television advertisements, stores, and online platforms (Table 2).

**Table 2:** Distribution of sources of knowledge about e-cigarettes, Jeddah, KSA, 2024 (*n* = 438)

	Number	Percent
**Source of knowledge about e-cigarettes**		
Parents	102	23.3
Friends and classmates	306	69.9
Newspapers	18	4.1
Magazines and television advertisements	25	5.7
Stores and supermarkets	38	8.7
Websites and forums	35	8.0
Not sure	20	4.6
**Total**	**438**	**100.0**

From all the participants, 52.5% classified e-cigarettes as electronic products. Regarding their effectiveness for smoking cessation, about half of the participants (50.5%) believed that e-cigarettes were completely ineffective, 29.7% believed it might help reduce smoking, 13.2% were not sure about their effect, and 6.6% considered them an effective method for quitting smoking. In terms of safety, 60.5% of the participants thought that e-cigarettes are equally harmful compared with traditional cigarettes, whereas 19.6% believed they are less harmful. (Table 3).

**Table 3:** Distribution of knowledge regarding e-cigarettes, Jeddah, KSA, 2024 (*n* = 438)

	Number	Percent
**What do you think about the nature of e-cigarettes?**		
Electronic products	230	52.5
Environmentally friendly alternative to traditional cigarettes	109	24.9
Smoking cessation products	50	11.4
Not sure	49	11.2
**Can e-cigarettes help you quit smoking?**		
Completely effective	29	6.6
Helping reduce smoking	130	29.7
Completely invalid	221	50.5
Not sure	58	13.2
**What’s your opinion about the health hazards of e-cigarettes compared to traditional cigarettes?**		
Completely healthy	25	5.7
Less harmful	86	19.6
Equally	265	60.5
Not sure	62	14.2

### Participants’ attitudes and perceptions towards e-cigarettes

Assessing the attitude of participants regarding e-cigarettes revealed that 45.9% of the participants stated that e-cigarettes may be more addictive than traditional cigarettes, while 29% thought that both smoking methods are equally addictive. 79.9% denied any role of e-cigarettes in preventing the harm of second-hand smoking. 71.92% of the participants agreed that e-cigarettes can cause cancer while 21.46% were not sure and only 6.62% believed that e-cigarettes are unrelated to cancer. About half (50.5%) of the participants felt that e-cigarettes could partially replace traditional cigarettes, and 91.6% thought that teenagers are more interested in e-cigarettes. Moreover, 49.2% of the participants believed that e-cigarettes may be a gateway to conventional smoking. The results showed that most participants stated that using e-cigarettes is a public health concern (48.9% agreed and 38.4% strongly agreed). In addition, 47% agreed and 27.9% strongly agreed that e-cigarettes should be regulated like other tobacco products, with 43.2% of the participants strongly agreed that e-cigarettes should be regulated in work and public places (Table 4).

**Table 4:** Distribution of attitudes towards e-cigarettes, Jeddah, KSA, 2024 (*n* = 438)

	Number	Percent
**What’s your opinion about the addiction to e-cigarettes when compared with traditional cigarettes?**		
Stronger than traditional cigarettes	201	45.9
Equally	127	29.0
Weaker than traditional cigarettes	69	15.8
No addiction	12	2.7
Not sure	29	6.6
**Do you think e-cigarettes could avoid the harm of second-hand smoke?**		
No	350	79.9
Yes	53	12.1
Not sure	35	8.0
**Do you think e-cigarettes have carcinogens?**		
No	29	6.62
Yes	315	71.92
Not sure	94	21.46
**Can e-cigarettes replace traditional cigarettes?**		
Completely	90	20.5
Partly	221	50.5
No	79	18.0
Not sure	48	11.0
**Do you think teenagers are more interested in e-cigarettes?**		
Yes	401	91.6
No	16	3.7
Not sure	21	4.8
**E-cigarette may be a gateway to conventional smoking**		
Strongly agree	20	4.6
Agree	189	43.2
Not sure	51	11.6
Disagree	56	12.8
Strongly disagree	122	27.9
**E-cigarette use is a public health concern?**		
Strongly agree	168	38.4
Agree	214	48.9
Not sure	28	6.4
Disagree	17	3.9
Strongly disagree	11	2.5
**E-cigarette should be regulated like other tobacco products**		
Strongly agree	122	27.9
Agree	206	47.0
Not sure	54	12.3
Disagree	34	7.8
Strongly disagree	22	5.0
**E-cigarettes should be regulated in work and public places?**		
Strongly agree	189	43.2
Agree	121	27.6
Not sure	50	11.4
Disagree	45	10.3
Strongly disagree	33	7.5

### Participants’ level of knowledge and attitude towards e-cigarettes

Overall, 39.3% of participants had a poor level of knowledge about e-cigarettes, while only 32.4% demonstrated good knowledge. In terms of attitude, 54.3% of Jeddah university students held a negative view of e-cigarettes, in contrast to 30.1% who maintained a positive attitude (Table 5).

**Table 5:** Distribution of the level of knowledge and attitudes towards e-cigarettes, Jeddah, KSA, 2024 (*n* = 438)

	Number	Percent
**Knowledge level**		
Good	142	32.4
Fair	124	28.3
Poor	172	39.3
**Attitude**		
Positive	132	30.1
Neutral	68	15.5
Negative	238	54.3

### Participants’ level of knowledge and attitudes towards e-cigarettes according to basic sociodemographic data

**Figure 1:**
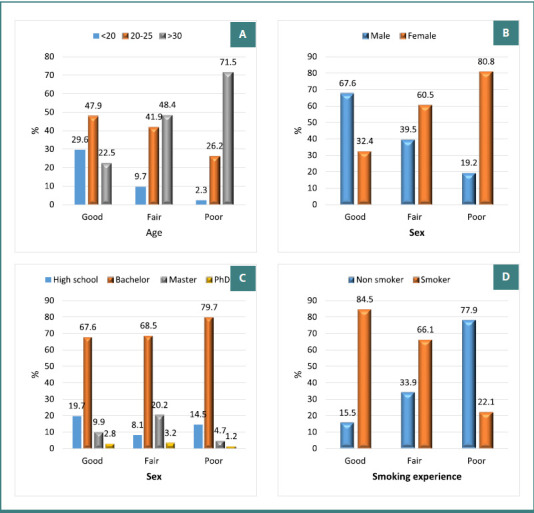
Relation between level of knowledge and basic sociodemographic data, Jeddah, KSA, 2024 (*n* = 438)

The study assessed the relationship between level of knowledge about e-cigarettes and basic sociodemographic data (Table 6). There was a significant difference between age groups; 71.5% of participants with good knowledge were in the 20–25-year age group, whereas 71.5% of those with poor knowledge were over 30 years old (*P* < 0.001; Figure 1A). Gender was also significantly associated with knowledge level (*P* < 0.001; Figure 1B). Among those with good knowledge, 67.6% were men. Conversely, among participants with poor knowledge, 80.8% were female, compared to only 19.2% men. Academic level was significantly associated with knowledge: among those with good knowledge, 19.7% were high school students, 67.6% held a bachelor’s degree, 9.9% were enrolled in a master’s program, and 2.8% had a PhD, while 79.7% of participants with poor knowledge held a bachelor’s degree (*P* < 0.002; Figure 1C). Furthermore, smoking history was significantly correlated with knowledge levels, with 84.5% of participants with good knowledge reporting a history of smoking compared to 77.9% in the poor knowledge group (*P* < 0.001; Figure 1D).

**Table 6:** Relationship between level of knowledge and basic sociodemographic data, Jeddah, KSA, 2024 (*n* = 438)

	Knowledge level						Total	X^2^ *P* value
	Good *n* = 142		Fair *n* = 124		Poor *n* = 172			
	No.	%	No.	%	No.	%		
**Age**								93.89 0.001*
<20	42	29.6	12	9.7	4	2.3	58	
20-25	68	47.9	52	41.9	45	26.2	165	
>30	32	22.5	60	48.4	123	71.5	215	
**Sex**								75.68 0.001*
Men	96	67.6	49	39.5	33	19.2	178	
Women	46	32.4	75	60.5	139	80.8	260	
**Level of education**								26.108 0.002*
High school	28	19.7	10	8.1	25	14.5	63	
Bachelor	96	67.6	85	68.5	137	79.7	318	
Master	14	9.9	25	20.2	8	4.7	47	
PhD	4	2.8	4	3.2	2	1.2	10	
**Smoking experience**								131.29 0.001*
Nonsmoker	22	15.5	42	33.9	134	77.9	198	
Smoker	120	84.5	82	66.1	38	22.1	240	

X^2^ = Chi square test; P was significant if ≤ 0.05; * Significant at level 0.05

**Figure 2:**
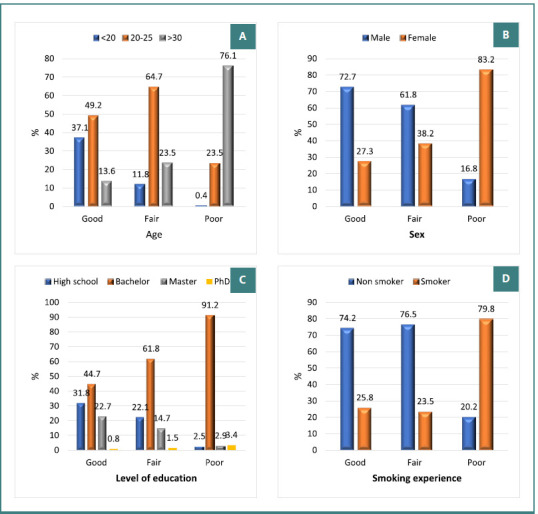
Relation between attitude and basic sociodemographic data, Jeddah, KSA, 2024 (*n* = 438)

The results of our questionnaire showed a significant difference in attitude levels between participants with different demographic characteristics (*P* <0.001; Table 7). The positive attitude towards e-cigarettes was higher among participants in the age group 20–35 years, followed by those who were less than 20 years and those over 30 years (65%, 49% and 18% respectively). On the other hand, a negative attitude was more prevalent among individuals over 30 years (76.1%), compared to 23.5% among those aged 20–35 years and 0.04% among participants younger than 20 years (Figure 2A). Male participants represented 72.7%, while female participants represented 27.3% of those with positive attitude. In addition, 83.2% of the participants with negative attitudes towards e-cigarettes were women while 16.8% were men (Figure 2B). Regarding the relationship between attitude and academic level, participants with bachelor’s degree expressed negative attitudes (91.2% of all participant with negative attitude) while most of participants with master degree showed positive attitudes (22.7% of all participants with positive attitude) (Figure 2C). 74.2% of participants with positive attitudes were non-smokers, while 79.8% of participants with negative attitudes were previous smokers (Figure 2D).

**Table 7:** Relationship between attitude and basic sociodemographic data, Jeddah, KSA, 2024 (*n* = 438)

	Attitude						Total	X^2^ *P* value
	Positive *n* = 132		Neutral *n* = 68		Negative *n* = 238			
	No.	%	No.	%	No.	%		
**Age**								95.11 0.001*
<20	49	37.1	8	11.8	1	0.4	58	
20-25	65	49.2	44	64.7	56	23.5	165	
>30	18	13.6	16	23.5	181	76.1	215	
**Sex**								124.95 0.001*
Male	96	72.7	42	61.8	40	16.8	178	
Female	36	27.3	26	38.2	198	83.2	260	
**Level of education**								115.449 0.001*
High school	42	31.8	15	22.1	6	2.5	63	
Bachelor	59	44.7	42	61.8	217	91.2	318	
Master	30	22.7	10	14.7	7	2.9	47	
PhD	1	0.8	1	1.5	8	3.4	10	
**Smoking experience**								131.998 0.001*
Non smoker	98	74.2	52	76.5	48	20.2	198	
Smoker	34	25.8	16	23.5	190	79.8	240	

X^2^ = Chi square test; *P* was significant if ≤ 0.05; * Significant at level 0.05

## Discussion

E-cigarettes are electronic devices designed to deliver nicotine and have gained popularity as a socially acceptable alternative to traditional tobacco smoking among young adults in Saudi Arabia [26]. This recent acceptance and popularity occur as a result of the misconception that e-cigarettes are a safe alternative for smokers with insufficient data about effective regulations to control its use [27]. These concerns lead us to conduct the current epidemiological study to have a better understanding of e-cigarette perceptions among college students in Jeddah, KSA.

### Awareness and knowledge about e-cigarettes

In this study, 438 students from different colleges in Jeddah city, located in the western region of KSA participated in the study. The prevalence of cigarette smoking among both undergraduate and post graduate students was 54.8%. This prevalence exceeds the previously reported prevalence of 14.1%, in the same geographic region of KSA [23]. Our study revealed that 43.8% of smokers had 3–6-year smoking experience with 52.1% smoking less than 10 cigarettes per day. These results were close to the results of a recent study [28] conducted in Saudi Arabia where about 40% reported that they were smoking for more than two years.

Although the availability of e-cigarettes in Saudi Arabia is a recent matter, our study showed that young students are exposed to information regarding e-cigarettes from friends and classmates (69.9%) or parents (23.3%) or other sources like social media (8%) and television (5.7%). Similar results were reported by Shehata *et al*. [29], who found that family and friends (53%) were the main source of e-cigarette information, followed by social media (51.9%) and mass media (34.6%). Similar results were also reported by Algassim *et al*. [30].

Regarding knowledge of e-cigarettes, 52.5% of participants classified e-cigarettes as electronic products, while a similar proportion also regarded them as environmentally friendly alternatives to traditional cigarettes. Another study [31] reported that 31.1% of respondents considered e-cigarettes to be tobacco products. In addition, our results found that 50.5% believed that e-cigarettes are completely ineffective in helping to quit smoking, 29.7% felt they might help reduce smoking, and only 6.6% considered them effective for quitting smoking. These results are in line with findings from other studies [29,30] that have evaluated the role of e-cigarettes in smoking cessation.

Focusing on the safety of e-cigarettes, 60.5% of the participants believed that e-cigarettes are equally harmful when compared with traditional cigarettes while 19.6% believed that e-cigarettes are less harmful. In a study among Mexican middle school students, 19% perceived e-cigarettes as less harmful than conventional smoking [32]. Similarly, a study conducted among students at Shaqra University in Saudi Arabia found that 34.9% thought e-cigarettes were safer than traditional cigarettes [30]. Therefore, additional studies are needed to find out whether e-cigarettes are safer than traditional nicotine cigarettes or whether both are equally dangerous.

### Attitude towards e-cigarettes

Our results assessing the attitude towards e-cigarettes found that 45.9% of Jeddah students believed that the addiction to e-cigarettes is stronger than traditional cigarettes and 29% stated equal addiction tendency. These findings contrast with another study [23] reporting that younger students believed traditional cigarettes to be more addictive, and with Qanash *et al*. [23], who found that 48.9% of respondents considered both regular and e-cigarettes equally addictive.

In our study, 79.9% of students rejected the notion that e-cigarette users can avoid the harm of secondhand smoke, aligning with evidence that e-cigarettes contribute to secondhand nicotine exposure [33]. In addition, 71.92% of participants thought that e-cigarettes have carcinogens. Previous research showed that 21.6% of the Shanghai University students in China, thought that e-cigarettes contain carcinogens [34]. Conversely, another study [35] suggested that e-cigarettes may be effective as a smoking cessation tool, with 39.3% of participants perceiving them as having a lower cancer risk than traditional cigarettes.

Similar to previous studies [36,37], our results found that 50.5% of students believed that e-cigarettes can partially replace traditional smoking. This may be explained by the belief that using e-cigarettes has a role in harm reduction or as a step towards cessation. On the other hand, 43.2% of the participants answered that e-cigarettes may be a gateway to traditional smoking initiation, which is in line with observational studies showing that smoking-naïve individuals who use e-cigarettes are more likely to become conventional cigarette smokers [38].

In addition, the majority of responses (91.6%) in the current study thought teenagers are more interested in e-cigarettes, a finding also reported by Pepper *et al*. [39]. This trend may be attributed to extensive exposure to e-cigarette advertising across various media platforms—including retail outlets, television, movies, the Internet, and print media—as well as the widespread use of social media, where approximately three-quarters of students have encountered e-cigarette-related content [40]. Additionally, the lower cost of e-cigarettes compared to traditional cigarettes may further contribute to their appeal among youth [41].

The Forum of International Respiratory Societies has recommended that e-cigarettes be regulated similarly to traditional tobacco products, including restrictions on media advertising, use in indoor and public places, and sale to adolescents and young adults [42]. A study recently conducted in Iraq [43] discussed the regulations applied for e-cigarettes use where 88.4% of participants believed that e-cigarettes use should be controlled in public places like regular cigarettes. Moreover, e-cigarettes are prohibited in Brazil [44]. In Saudi Arabia, a study conducted in 2021 [30] found that 73.4% of respondents believed that e-cigarettes should be restricted in public areas. Similarly, our results revealed that the majority of students believed that e-cigarettes use is a public health concern and should be controlled like other tobacco products in public as well as the workplace.

Although nearly all participants were aware of e-cigarettes, knowledge regarding their hazardous effects was insufficient, with 39.3% demonstrating poor knowledge and only 30.1% expressing a positive attitude toward e-cigarettes. These results are comparable to a study among university students in Riyadh, KSA, in which 48.4% agreed that e-cigarettes contain similar harmful substances to traditional cigarettes [45]. Generally, there was a knowledge gap (17.1%) regarding official regulations and other details regarding e-cigarettes.

### Level of knowledge and attitude according to sociodemographic and personal characteristics

Regarding the level of knowledge about e-cigarettes, our findings indicate that only 22.1% of students with poor knowledge were smokers, whereas 77.9% of those with good knowledge were non-smokers. These findings contrast with a study conducted in Pakistan [46], which reported a positive correlation between smoking experience and better knowledge of e-cigarettes. In addition, significant differences in attitudes were observed between smokers and non-smokers; 74.2% of participants with a positive attitude toward e-cigarettes were smokers, while 79.8% of those with a negative attitude were non-smokers.

There was a significant gender difference in knowledge and attitudes towards e-cigarettes, with 80.8% of those with poor level of knowledge and 83.2% of those with negative attitudes being women. This result differs somewhat from a previous study [24], in which most female participants demonstrated better knowledge of e-cigarettes and expressed stronger disapproval of their use. Age was also significantly associated with knowledge and attitude. In the current study, 71.5% of participants with good knowledge were aged 20–25 years, while 71.5% of those with poor knowledge were over 30 years. Similarly, a positive attitude toward e-cigarettes was most prevalent among participants aged 20–35 years, followed by those under 20 years, with the lowest positive attitude observed in those over 30 years. These findings contrast with recent research [24], which found that participants aged 25 years or older had poorer knowledge, and that younger participants were more likely to use e-cigarettes despite a higher negative attitude among those over 30.

Academic level was significantly related to both knowledge and attitude. Among participants with good knowledge, 19.7% were high school students and 67.6% held a bachelor’s degree. In contrast, 79.7% of those with poor knowledge held a bachelor’s degree. In terms of attitude, 91.2% of participants with a negative attitude possessed a bachelor’s degree, whereas 22.7% of those with a positive attitude had attained a master’s degree. These results are comparable to previous studies [24,46].

### Limitations

We recognize that our study is limited by its focus on a specific population characterized by a high level of education and specific geographic location, thus limiting the applicability of the study results to the broader population of Saudi Arabia. Moreover, the reliance on the questionnaire and self-reported data increases potential recall biases as well as social desirability. Therefore, conducting a larger study on Saudi population from different regions of the KSA as well as diverse social and educational sectors will have more comprehensive insights.

## CONCLUSION

According to our findings, although overall awareness of e-cigarettes was generally acceptable, there was a significant prevalence of poor knowledge and a misleadingly positive attitude toward these products. Alarmingly, notable knowledge gaps were identified. The results highlight an urgent need for targeted educational initiatives addressing the health risks and safety profile of electronic smoking. This need is particularly critical given the rising use of e-cigarettes, especially among young adults and university students—predominantly males and individuals with no prior history of smoking.

## Data Availability

Further data is available from the corresponding author upon reasonable request.

## Personal thanks

We deeply acknowledge all the participants for their valuable participation in the study.
